# Total Synthesis of Cyclopiamide A Using Palladium-Catalyzed Domino Cyclization

**DOI:** 10.3390/molecules25214903

**Published:** 2020-10-23

**Authors:** Sunhwa Park, Kye Jung Shin, Jae Hong Seo

**Affiliations:** Integrated Research Institute of Pharmaceutical Sciences, College of Pharmacy, The Catholic University of Korea, Bucheon-si, Gyeonggi-do 420-743, Korea; sunalovegs@naver.com (S.P.); kyejung@catholic.ac.kr (K.J.S.)

**Keywords:** cyclopiamide A, domino reaction, palladium-catalyzed, carbopalladation, C-H activation

## Abstract

Total synthesis of cyclopiamide A was accomplished using a palladium-catalyzed domino cyclization. Three rings in the tetracyclic skeleton of cyclopiamide A were constructed in a one-step domino reaction incorporating double carbopalladation and C-H activation.

## 1. Introduction

**Figure 1:**
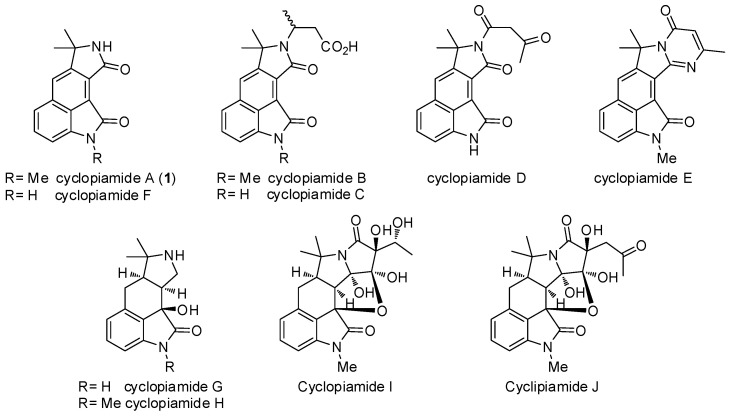
Cyclopiamide A (**1**) and its derivatives.

Cyclopiamide A (**1**) was first isolated from *Penicillium cyclopium* in 1990 by Holzapfel and coworkers (Figure 1) [1]. The isolation of nine cyclopiamide derivatives (cyclopiamide B–J) was recently reported [2,3]. All of the isolated compounds showed moderate toxicity (LD_50_ = 14.1~38.5 μg/mL) in brine shrimp assays. Despite the unique tetracyclic skeleton of cyclopiamide A (**1**), synthetic efforts for **1** were rarely reported. In 2018, the first and only report of total synthesis of cyclopiamide A (**1**) was published by Wood and coworkers [4]. As part of our ongoing efforts to develop novel synthetic methods for 3-methyleneoxindole derivatives [5,6,7,8,9], we describe herein the synthesis of cyclopiamide A (**1**) featuring rapid and efficient construction of the tetracyclic ring system via a palladium-catalyzed domino reaction.

**Scheme 1:**
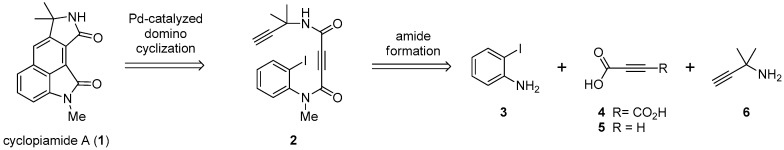
Retrosynthetic analysis of cyclopiamide A (**1**).

Retrosynthetically, we hypothesized that the tetracyclic system of cyclopiamide A (**1**) may be accessed from diyne **2** via palladium-catalyzed domino cyclization (Scheme 1). The key intermediate **2** would be synthesized by amide formation between amine groups of aniline **3** and propargylamine **6**, and acetylenedicarboxylic acid (**4**) or propiolic acid (**5**).

## 2. Results and Discussion

**Scheme 2:**
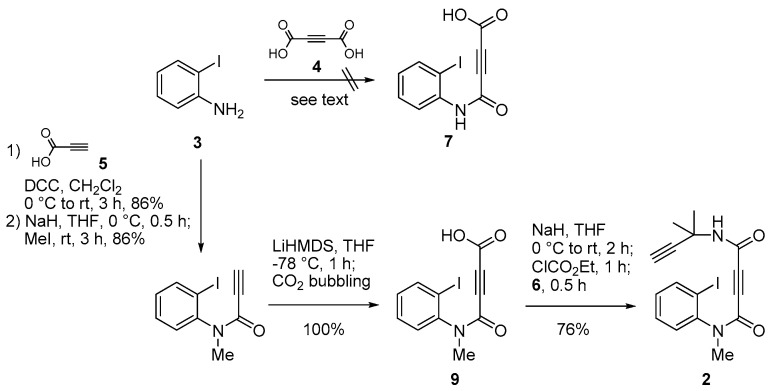
Preparation of the key intermediate diyne **2**.

Our total synthesis of cyclopiamide A (**1**) began with the synthesis of the key intermediate diyne **2** (Scheme 2). We first examined the feasibility of monoamide formation between acetylenedicarboxylic acid (**4**) and aniline **3**. The synthesis of monoamides from **4** has been only rarely reported and reaction yields were poor [10]. However, several synthetic methods for creating a symmetric diamide from **4** were recently reported using EDCI (1-ethyl-3-(3-dimethylamino-propyl)carbodiimide) and DMTMM (4-(4,6-dimethoxy-1,3,5-triazin-2-yl)-4-methyl-morpholinium chloride) [11,12]. We therefore examined coupling reactions with EDCI and DMTMM, along with other general coupling reagents (DCC (*N*,*N*′-dicyclohexylcarbodiimide) and PyBOP (benzotriazol-1 -yl-oxytripyrrolidinophosphonium hexafluorophosphate)). Unfortunately, none of these reactions afforded the desired amide **7**, probably due to competing decarboxylation and/or 1,4-addition, which are known side reactions in the formation of amides/esters from acetylenedicarboxylic acid [13]. Reactions incorporating other derivatives of **4**, such as the monoester, acid chloride, and mixed anhydride, were also evaluated, but none yielded **7**. Even when the coupling amine was changed from aniline **3** to propargylamine **6**, the corresponding monoamide was not produced. We then used Overman’s stepwise approach [14] to synthesize the unsymmetric diamide of **4**. Although this approach took longer, it was a more robust way of creating diyne **2**. Thus, the known propiolamide **8** was prepared by DCC coupling of aniline **3** and propiolic acid (**5**), followed by methylation of the corresponding amide with NaH and MeI [15]. The carboxylic acid group was successfully introduced to **8** using Overman’s method (lithiation of the terminal alkyne and CO_2_ quenching) to afford monoamide **9**. Formation of the second amide was more problematic than expected, since Overman’s EDCI coupling condition was not effective with our substrate **9**, giving only trace amounts of the desired diyne **2**. After testing several coupling conditions, we found that Roussi’s mixed anhydride method [16] was suitable for our purposes. Thus, carboxylic acid **9** was transformed to the corresponding mixed anhydride using a strong metal base (NaH) and ClCO_2_Et. Addition of propargylamine **6** to the mixed anhydride produced the desired diyne **2** in 76% yield.

**Scheme 3:**
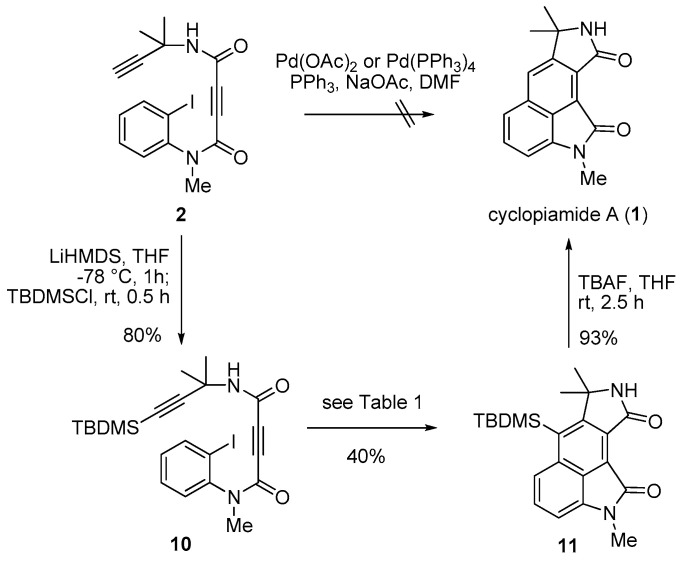
Domino cyclization and total synthesis of cyclopiamide A (**1**).

Using the key intermediate diyne **2**, we determined the conditions needed for palladium-catalyzed domino cyclization of **2** to complete the total synthesis of cyclopiamide A (**1**) (Scheme 3). Under several of the evaluated sets of conditions, desired product **1** was not produced within an unidentifiable mixture of by-products. A literature search for similar domino intramolecular carbopalladation systems ending with the C-H activation of alkyne substrates revealed that an internal alkyne is essential for successful transformation [17,18,19,20,21,22]. Therefore, the terminal alkyne group of **2** was protected with a silyl group using LiHMDS and TBDMSCl to obtain the internal alkyne **10** in 80% yield. Our efforts to optimize the domino cyclization conditions to obtain **10** are detailed in Table 1. Under our optimized conditions, diyne **10** was smoothly transformed into the tetracyclic compound **11** in 40% yield. The total synthesis of cyclopiamide A (**1**) was completed after deprotection of the TBDMS group of **11** by TBAF (93% yield).

Initially, diyne **10** was exposed to general conditions [Pd(OAc)_2_ (10 mol%), PPh_3_ (20 mol%), NaOAc (3.0 eq), DMF, 60 °C, 3 h], which afforded the cyclized product **11** as the only isolable product in 29% yield (Table 1, entry 1). Raising or lowering the reaction temperature did not improve the yield, but did affect the reaction rate (Entries 2 and 3). To examine ligand effects, the reaction was performed without ligand (Entry 4) and with other phosphine ligands (Entries 5–7). None of these reactions gave greater yields than that obtained with PPh_3_. When the amount of PPh_3_ was increased to 30 mol%, the yield of the reaction increased only slightly to 31% (Entry 8). Several other bases were also evaluated, but all gave similar or inferior results (Entries 9–11). Changing the catalyst from Pd(OAc)_2_ to Pd(PPh_3_)_4_ resulted in a similar yield of 30% (Entry 12). Interestingly, in the case of Pd(PPh_3_)_4_, the reaction without phosphine ligand provided **11** in a slightly higher yield (35%) (Entry 13). Increasing the catalyst load to 20 mol% afforded the desired cyclized product **11** in 40% yield (Entry 14).

**Scheme 4:**
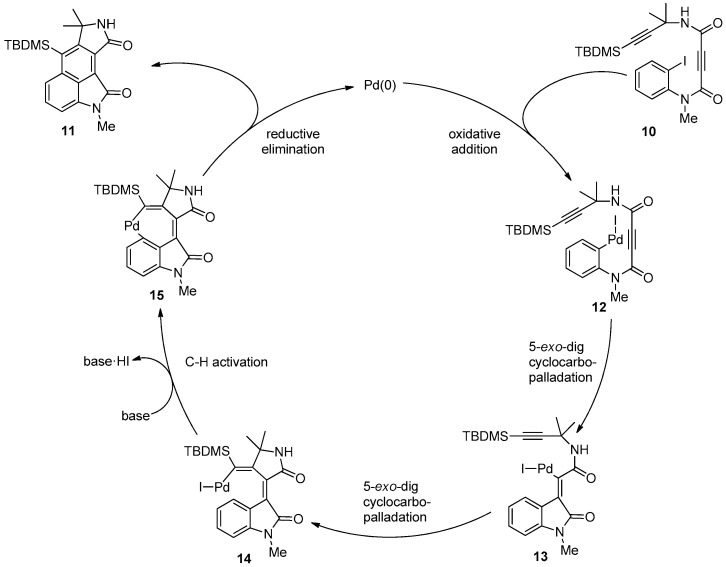
Proposed catalytic cycle for domino cyclization.

Mechanistically, our palladium-catalyzed domino reaction can be depicted as shown in Scheme 4. Oxidative addition of an active Pd(0) catalyst into C-I bond of **10** gives the aryl palladium intermediate **12**, which is converted in turn to **13** and **14** through double 5-*exo*-dig cyclocarbopalladations. The proximity of Pd and aryl C-H in **14** facilitates C-H activation to give intermediate **15**, which is then transformed into **11** by reductive elimination.

## 3. Experimental Section 

### 3.1. General Information

All reactions were performed under an argon atmosphere with dry solvents, unless otherwise stated. Dry tetrahydrofuran (THF) and methylene chloride (CH_2_Cl_2_) were obtained from Ultimate Solvent Purification System (JC Meyer Solvent System, Laguna Beach, CA, USA). Other dry solvents were purchased as anhydrous grade. All commercially available reagents were purchased and used without further purification. Reactions were monitored by thin-layer chromatography (TLC) on silica gel plates (Merck TLC Silica Gel 60 F254, Darmstadt, Germany) using UV light, PMA (an ethanolic solution of phosphomolybdic acid), or ANIS (an ethanolic solution of para-anisaldehyde) as visualizing agent. Purification of products was conducted by column chromatography through silica gel 60 (0.060−0.200 mm). Melting points of all solid compounds were determined by Buchi M-565. IR spectra were recorded on a Jasco P-2000 FT-IR spectrometer (JASCO Inc., Easton, MD, USA). NMR spectra were obtained on Bruker AVANCE III 500 MHz (Bruker Corporation, Billerica, MA, USA) using residual undeuterated solvent or TMS (tetramethylsilane) as an internal reference. Copies of all NMR spectra are provided as Supplementary Materials for this article. High-resolution mass spectra (HR-MS) were recorded on a Agilant 6530 Q-TOF (Agilant, Santa Clara, CA, USA) using FAB (fast atom bombardment) or a JEOL JMS-700 (JEOL, Tokyo, Japan) using EI (electron impact).

### 3.2. Synthesis of ***1***,***2*** and ***8***–***11***

*N-(2-Iodophenyl)-N-methylpropiolamide* (**8**) [15,23]: To a stirred solution of 2-iodoaniline (**3**) (500 mg, 2.28 mmol, 1.0 equiv.) in CH_2_Cl_2_ (22 mL) and propiolic acid (**5**) (0.18 mL, 2.97 mmol, 1.3 equiv.) was added dicyclohexylcarbodimide (DCC, 612 mg, 2.97 mmol, 1.3 equiv.) at 0 °C. Then, the temperature was gradually raised to 25 °C over 30 min. The mixture was stirred at the same temperature for 3 h and diluted with EtOAc (100 mL). Organic layer was washed with 2 M aqueous HCl (30 mL × 2) and sat. aqueous NaHCO_3_ (30 mL × 2). The organic layer was separated, dried (Na_2_SO_4_), filtered and concentrated under reduced pressure. The crude residue was purified by column chromatography (silica gel, hexanes:EtOAc = 6:1) to give *N*-(2-iodophenyl)propiolamide [15,23] (531 mg, 86%) as a white solid (mp = 103.7 °C). IR (film) 3288, 2107, 1662, 1515, 1432 cm^−1^; ^1^H NMR (500 MHz, CDCl_3_): δ = 8.18 (d, *J* = 8.2 Hz, 1H), 7.82 (brs, 1H), 7.80 (d, *J* = 7.9 Hz, 1H), 7.36 (t, *J* = 7.8 Hz, 1H), 6.89 (t, *J* = 7.6 Hz, 1H), 3.01 (s, 1H) ppm; ^13^C NMR (125 MHz, CDCl_3_): δ = 149.7, 139.1, 137.5, 129.5, 126.9, 122.5, 89.6, 77.5, 74.8 ppm.; To a stirred suspension of NaH (60% in mineral oil, 66.0 mg, 1.66 mmol, 1.1 equiv.) in THF (7.0 mL) was added a solution of the previously prepared *N*-(2-iodophenyl)-propiolamide (408 mg, 1.51 mmol, 1.0 equiv.) in THF (8.0 mL) at 0 °C. After 30 min stirring, MeI (103 μL, 1.66 mmol, 1.1 equiv.) was added dropwise at the same temperature. Then, the temperature was gradually raised to 25 °C. The mixture was stirred for additional 3 h. The solvent was removed under reduced pressure. The residue was diluted with EtOAc (100 mL) and washed with sat. aqueous NH_4_Cl (30 mL × 2). The organic layer was dried (Na_2_SO_4_), filtered, and concentrated under reduced pressure. The crude residue was purified by column chromatography (silica gel, hexanes:EtOAc = 4:1) to give *N*-methylpropiolamide **8** (369 mg, 86%) as a white solid (mp = 101.0 °C (lit. [24] 101–102 °C)). IR (film) 3213, 2103, 1633, 1467, 1377 cm^−1^; ^1^H NMR (500 MHz, CDCl_3,_ 8:1 atropisomeric mixture, major peaks): δ = 7.93 (dd, *J* = 7.9, 1.3 Hz, 1H), 7.43 (ddd, *J* = 8.7, 7.6, 1.3 Hz, 1H), 7.32 (dd, *J* = 7.8, 1.6 Hz, 1H), 7.12 (ddd, *J* = 9.1, 7.7, 1.6 Hz, 1H), 3.23 (s, 3H), 2.74 (s, 1H) ppm; ^13^C NMR (125 MHz, CDCl_3_): δ = 153.2, 145.1, 140.1, 130.5, 129.8, 129.7, 99.5, 79.1, 76.3, 35.5 ppm.

*4-((2-Iodophenyl)(methyl)amino)-4-oxobut-2-ynoic acid* (**9**): To a solution of *N*-methylpropiolamide **8** (369 mg, 1.29 mmol, 1.0 equiv.) in THF (12 mL) was added LiHMDS (1.0 M in THF, 1.55 mL, 1.55 mmol, 1.2 equiv.) dropwise at −78 °C. The resulting solution was stirred at −78 °C for 1 h. Then, CO_2_ gas (dry ice) was bubbled through the solution for 30 min. Then, the reaction flask was opened and allowed to warm to room temperature. The solvent was removed under reduced pressure. To the residue H_2_O (30 mL) and hexane (15 mL) were added. The aqueous layer was separated, acidified with 2 M aqueous HCl to pH 1~2 and then extracted with EtOAc (20 mL × 3). The organic layers were combined, dried (Na_2_SO_4_), filtered and concentrated under reduced pressure to give acid **9** (425 mg, 100%) as off white solid (mp = 125.4 °C). The crude **9** was used for the next step without further purification. IR (film) 3057, 2924, 1719, 1650, 1265, 735 cm^−1^; ^1^H NMR (500 MHz, CDCl_3,_ 7:1 atropisomeric mixture, major peaks): δ = 8.85 (brs, 1H), 7.94 (dd, *J* = 7.9, 1.1 Hz, 1H), 7.45 (td, *J* = 7.7, 1.2 Hz, 1H), 7.33 (dd, *J* = 7.8, 1.4 Hz, 1H), 7.15 (td, *J* = 7.8, 1.4 Hz, 1H), 3.26 (s, 3H) ppm; ^13^C NMR (125 MHz, CDCl_3_): δ = 153.7, 152.6, 143.6, 140.4, 131.2, 130.1, 129.5, 98.9, 80.2, 77.2, 36.0 ppm; HRMS (FAB): calcd. for C_11_H_9_INO_3_ [M + H^+^]: 329.9627, found 329.9625.

*N^1^-(2-Iodophenyl)-N^1^-methyl-N^4^-(2-methylbut-3-yn-2-yl)but-2-ynediamide* (**2**): To a stirred suspension of NaH (60% in mineral oil, 76.0 mg, 1.91 mmol, 1.5 equiv.) in THF (5.0 mL) was added a solution of acid **9** (420 mg, 1.28 mmol, 1.0 equiv.) in THF (7.0 mL) at 0 °C. The reaction mixture was slowly warmed up to room temperature and stirred for 2 h. After addition of ethyl chloroformate (180 μL, 1.91 mmol, 1.5 equiv.), the reaction mixture was stirred for 1 h. And then 1,1-dimethylpropargylamine (**6**) (107 μL, 1.02 mmol, 0.8 equiv.) was added and stirring was continued for another 30 min. The solvent was removed under reduced pressure and the residue was diluted with EtOAc (50 mL). The mixture was washed with sat. aqueous NaHCO_3_ (20 mL × 2) and the organic layer was dried (Na_2_SO_4_), filtered, and concentrated under reduced pressure. The crude residue was purified by column chromatography (silica gel, hexanes:EtOAc = 2:1) to give diyne **2** (308 mg, 76%) as a white solid (mp = 123.5 °C). IR (film) 3294, 3260, 1662, 1626, 1529, 1387, 1278 cm^−1^; ^1^H NMR (500 MHz, CDCl_3,_ 6:1 atropisomeric mixture): δ = 7.95 (dd, *J* = 8.0, 1.3 Hz, 1H, *major*), 7.91 (dd, *J* = 8.0, 1.3 Hz, 1H, *minor*), 7.46 (td, *J* = 7.7, 1.4 Hz, 1H, *major*), 7.42 (td, *J* = 7.7, 1.4 Hz, 1H, *minor*), 7.33 (dd, *J* = 7.8, 1.5 Hz, 1H, *major*), 7.21 (dd, *J* = 7.8, 1.5 Hz, 1H, *minor*), 7.14 (td, *J* = 7.8, 1.6 Hz, 1H, *major*), 7.07 (td, *J* = 7.7, 1.5 Hz, 1H, *minor*), 6.27 (brs, 1H, *minor*), 5.78 (brs, 1H, *major*), 3.50 (s, 3H, *minor*), 3.25 (s, 3H, *major*), 2.42 (s, 1H, *minor*), 2.34 (s, 1H, *major*), 1.69 (s, 6H, *minor*), 1.57 (s, 6H, *major*) ppm; ^13^C NMR (125 MHz, CDCl_3_): δ = 152.6 (*major*), 152.4 (*minor*), 150.1 (*minor*), 149.8 (*major*), 144.2 (*major*), 143.5 (*minor*), 140.3 (*major*), 140.2 (*minor*), 131.0 (*major*), 130.2 (*minor*), 130.0 (*major*), 129.7 (*major*), 128.4 (*minor*), 99.2 (*major*), 97.3 (*minor*), 85.83 (*major*), 85.78 (*minor*), 83.3 (*minor*), 82.5 (*major*), 74.4 (*major*), 74.1 (*minor*), 70.4 (*minor*), 70.2 (*major*), 49.0 (*minor*), 48.9 (*major*), 39.3 (*minor*), 35.8 (*major*), 28.8 (*minor*), 28.70 (*major*), 28.68 (*major*) ppm; HRMS (FAB): calcd. for C_16_H_16_IN_2_O_2_ [M + H^+^]: 395.0256, found 395.0259.

*N^1^-(4-(tert-Butyldimethylsilyl)-2-methylbut-3-yn-2-yl)-N^4^-(2-iodophenyl)-N^4^-methylbut-2-ynediamide* (**10**): To a stirred solution of diyne **2** (295 mg, 0.748 mmol, 1.0 equiv.) in THF (7.5 mL) was added LiHMDS (1.0 M in THF, 2.24 mL, 2.24 mmol, 3.0 equiv.) at −78 °C. After 1 h TBDMSCl (338 mg, 2.24 mmol, 3.0 equiv.) was added and the temperature was gradually raised to room temperature. The mixture was stirred at room temperature for 30 min and the solvent was removed under reduced pressure. The residue was diluted with EtOAc (50 mL) and was washed with H_2_O (20 mL × 2). The organic layer was dried (Na_2_SO_4_), filtered and concentrated under reduced pressure. The crude residue was purified by column chromatography (silica gel, hexanes:EtOAc = 3:1) to give TBDMS-protected diamide **10** (304 mg, 80%) as a white solid (mp = 101.1 °C). IR (film) 3261, 1678, 1644, 1535, 1470, 830 cm^−1^; ^1^H NMR (500 MHz, CDCl_3,_ 6:1 atropisomeric mixture): δ = 7.94 (dd, *J* = 7.9, 1.1 Hz, 1H, *major*), 7.91 (dd, *J* = 8.0, 1.1 Hz, 1H, *minor*), 7.45 (td, *J* = 7.7, 1.2 Hz, 1H, *major*), 7.42 (td, *J* = 7.9, 1.1 Hz, 1H, *minor*), 7.33 (dd, *J* = 7.8, 1.4 Hz, 1H, *major*), 7.21 (dd, *J* = 7.9, 1.4 Hz, 1H, *minor*), 7.14 (td, *J* = 7.8, 1.5 Hz, 1H, *major*), 7.08 (td, *J* = 7.8, 1.5 Hz, 1H, *minor*), 6.21 (brs, 1H, *minor*), 5.77 (brs, 1H, *major*), 3.50 (s, 3H, *minor*), 3.25 (s, 3H, *major*), 1.69 (s, 6H, *minor*), 1.57 (s, 6H, *major*), 0.94 (s, 9H, *minor*), 0.91 (s, 9H, *major*), 0.11 (s, 6H, *minor*), 0.08 (s, 6H, *major*) ppm; ^13^C NMR (125 MHz, CDCl_3_): δ = 152.7 (*major*), 152.4 (*minor*), 149.8 (*minor*), 149.5 (*major*), 144.3 (*major*), 143.5 (*minor*), 140.30 (*minor*), 140.28 (*major*), 130.9 (*major*), 130.3 (*minor*), 130.04 (*minor*), 130.02 (*major*), 129.7 (*major*), 128.4 (*minor*), 108.6 (*major*), 108.3 (*minor*), 99.2 (*major*), 97.3 (*minor*), 85.0 (*minor*), 84.8 (*major*), 83.5 (*minor*), 82.7 (*major*), 74.1 (*major*), 73.9 (*minor*), 50.4 (*minor*), 50.2 (*major*), 39.3 (*minor*), 35.7 (*major*), 28.7 (*minor*), 28.5 (*major*), 28.4 (*major*), 26.18 (*minor*), 26.17 (*major*), 16.7 (*minor*), 16.6 (*major*), −4.57 (*minor*), −4.59 (*major*) ppm; HRMS (FAB): calcd. for C_22_H_30_IN_2_O_2_Si [M + H^+^]: 509.1121, found 509.1118.

*6-(tert-Butyldimethylsilyl)-2,7,7-trimethyl-7,8-dihydro-1H-isoindolo[4,5,6-cd]indole-1,9(2H)-dione* (**11**): To a solution of TBDMS-protected diamide **10** (50.0 mg, 98.3 μmol, 1.0 equiv.) in DMF (2.0 mL) was added Pd(PPh_3_)_4_ (22.6 mg, 19.7 μmol, 20 mol%) and NaOAc (24.0 mg, 295 μmol, 3.0 equiv.). The reaction mixture was stirred at 60 °C for 4.5 h, and then cooled to room temperature. The mixture was diluted with EtOAc (100 mL) and washed with H_2_O (10 mL × 3). The organic layer was then dried (Na_2_SO_4_), filtered and concentrated under reduced pressure. The crude residue was purified by column chromatography (silica gel, CH_2_Cl_2_:MeOH = 15:1) to give **11** (15.0 mg, 40%) as an orange solid (mp = 309.1 °C). IR(film) 3037, 2854, 2352, 1721, 1627, 1187 767, 687 cm^−1^; ^1^H NMR (500 MHz, CDCl_3_): δ = 8.02 (brs, 1H), 7.95 (d, *J* = 9.0 Hz, 1H), 7.46 (dd, *J* = 9.0, 7.0 Hz, 1H), 6.84 (d, *J* = 7.0 Hz, 1H), 3.44 (s, 3H), 1.82 (s, 6H), 0.97 (s, 9H), 0.77 (s, 6H) ppm; ^13^C NMR (125 MHz, CDCl_3_): δ = 165.7, 165.3, 159.5, 142.0, 137.3, 137.2, 129.6, 128.3, 125.6, 124.8, 124.0, 104.0, 62.2, 29.8, 29.5, 26.4, 19.2, 5.6 ppm; HRMS (EI): calcd. for C_22_H_28_N_2_O_2_Si [M^+^]: 380.1920, found 380.1921.

*Cyclopiamide A* (**1**): To a stirred solution of **11** (14.0 mg, 36.8 μmol, 1.0 equiv.) in THF (1 mL) was added TBAF (1.0 M solution in THF, 0.15 mL, 0.15 mmol, 4.0 equiv.) at room temperature. The reaction mixture was stirred for 2.5 h, diluted with EtOAc (20 mL) and washed with sat. aqueous NH_4_Cl (10 mL × 2). Organic layer was dried (Na_2_SO_4_), filtered and concentrated under reduced pressure. The crude residue was purified by column chromatography (silica gel, CH_2_Cl_2_:MeOH = 15:1) to give cyclopiamide A (**1**) (9.1 mg, 93%) as a yellow solid (mp = 251.8 °C). IR (film) 3519, 3072, 1707, 1636, 1497, 1390, 1046, 768 cm^−1^; ^1^H NMR (500 MHz, CDCl_3_): δ = 7.97 (s, 1H), 7.55 (m, 2H), 7.16 (brs, 1H), 6.88 (m, 1H), 3.46 (s, 3H), 1.68 (s, 6H) ppm; ^13^C NMR (125 MHz, CDCl_3_): δ = 166.7, 165.5, 153.6, 141.7, 131.0, 130.4, 129.2, 125.8, 124.2, 122.8, 120.2, 104.6, 59.9, 28.8, 26.5 ppm; HRMS (FAB): calcd. for C_16_H_15_N_2_O_2_ [M + H^+^]: 267.1134, found 267.1137.

## 4. Conclusions

The total synthesis of cyclopiamide A (**1**) was accomplished with an overall yield of 16.7% using a concise seven-step method. The tetracyclic skeleton of **1** was efficiently obtained in a single step through a palladium-catalyzed domino reaction combining double cyclocarbopalladation and C-H activation. We are currently exploring the synthesis of other cyclopiamide derivatives using this strategy.

## Figures and Tables

**Table 1:**
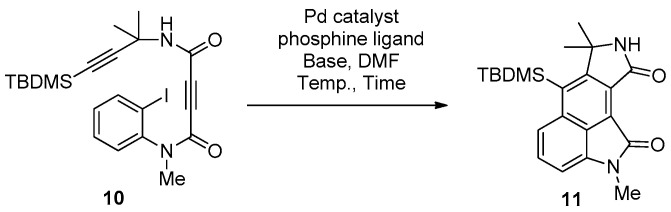
Optimization of domino cyclization reaction of **10** ^1^.

Entry	Pd Catalyst	Phosphine Ligand	Base	Temp (°C)	Time (h)	Yield (%) ^2^
1	Pd(OAc)_2_	PPh_3_	NaOAc	60	3	29
2	Pd(OAc)_2_	PPh_3_	NaOAc	rt	24	23
3	Pd(OAc)_2_	PPh_3_	NaOAc	100	1	27
4	Pd(OAc)_2_	–	NaOAc	60	2	20
5	Pd(OAc)_2_	dppf	NaOAc	60	2	29
6	Pd(OAc)_2_	P(*o*-tol)_3_	NaOAc	100	21	18
7 ^3^	Pd(OAc)_2_	*t*-BuXPhos	NaOAc	60	3	21
8 ^3^	Pd(OAc)_2_	PPh_3_	NaOAc	60	3.5	31
9 ^3^	Pd(OAc)_2_	PPh_3_	K_2_CO_3_	60	3	24
10 ^3^	Pd(OAc)_2_	PPh_3_	K_3_PO_4_	60	4	31
11 ^3^	Pd(OAc)_2_	PPh_3_	Cs_2_CO_3_	60	3	16
12 ^3^	Pd(PPh_3_)_4_	PPh_3_	NaOAc	60	4	30
13	Pd(PPh_3_)_4_	–	NaOAc	60	4	35
14 ^4^	Pd(PPh_3_)_4_	–	NaOAc	60	4.5	40

^1^ Reaction conditions: 10 (1.0 eq), Pd catalyst (10 mol%), phosphine ligand (20 mol%), base (3 eq), DMF (0.05 M), temp (°C), time (h). ^2^ Isolated yield. ^3^ 30 mol% of phosphine ligand was used. ^4^ 20 mol% of Pd catalyst was used.

## Supplementary Materials

The following are available online. Copies of NMR spectra of **1**,**2** and **8**–**11**.
