# Supplementary material for: Total Synthesis of Cyclopiamide A Using Palladium-Catalyzed Domino Cyclization
Source: Molecules. 2020 Oct 23;25(21):4903. doi: 10.3390/molecules25214903 (PMC7660198; doi:10.3390/molecules25214903)
Supplement: Supplementary file 1 [file molecules-25-04903-s001.pdf]

<*Supporting Information*>

Total Synthesis of Cyclopiamide A Utilizing A Palladium-Catalyzed Domino Cyclization

Sunhwa Park, Kye Jung Shin, Jae Hong Seo*

Integrated Research Institute of Pharmaceutical Sciences, College of Pharmacy, The Catholic University of Korea, 43 Jibong-ro, Wonmi-gu, Bucheon, Gyeonggi-do 14662, Republic of Korea

E-mail: jaehongseo@catholic.ac.kr

Contents

^{1}H and ^{13}C NMR spectra of *N*-(2-iodophenyl)propiolamide **2**

^{1}H and ^{13}C NMR spectra of **8** **3**

^{1}H and ^{13}C NMR spectra of **9** **4**

^{1}H and ^{13}C NMR spectra of **2** **5**

^{1}H and ^{13}C NMR spectra of **10** **6**

^{1}H, ^{13}C, HSQC and HMBC NMR spectra of **11** **7**

^{1}H, ^{13}C, HSQC and HMBC NMR spectra of cyclopiamide A (**1**) **9**

Tabulated spectral comparison of ^{1}H and ^{13}C NMR spectra between synthetic and natural cyclopiamide A...... **11**

N-(2-Iodophenyl)propiolamide

^{1}H NMR spectrum

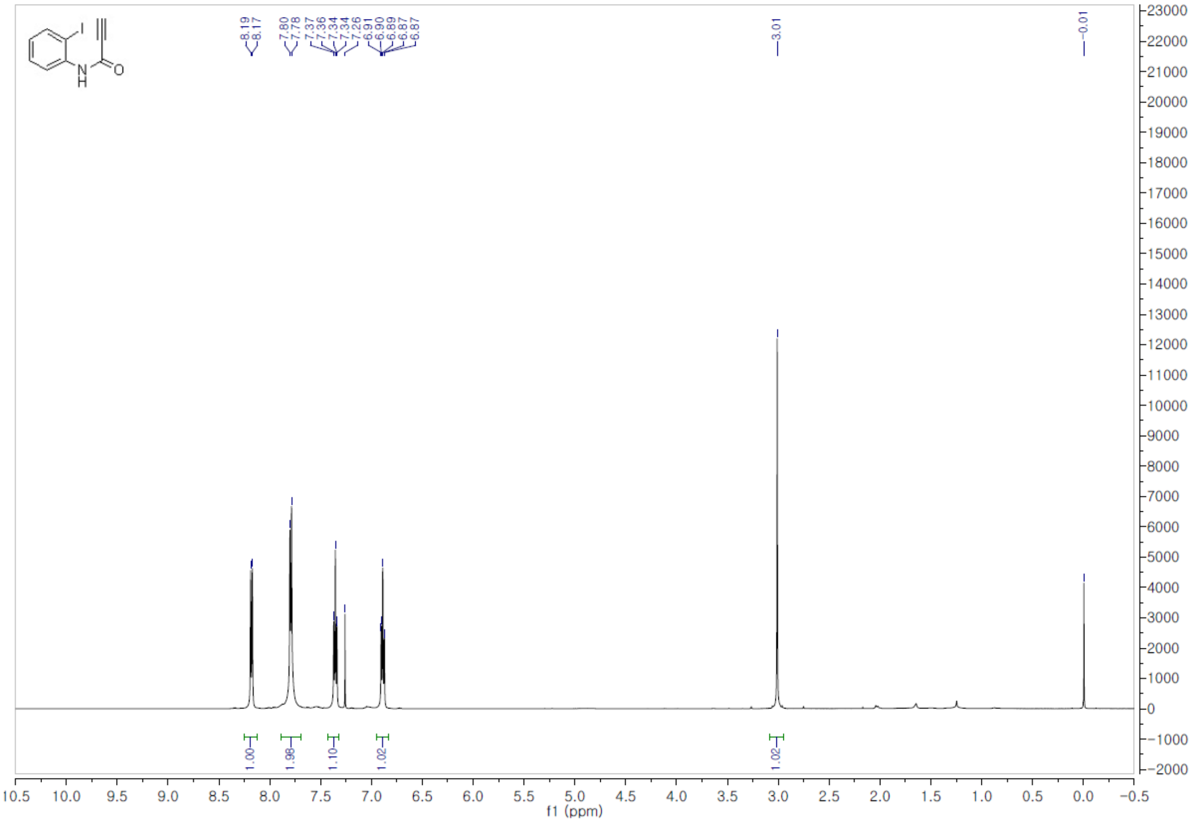

^{13}C NMR spectrum

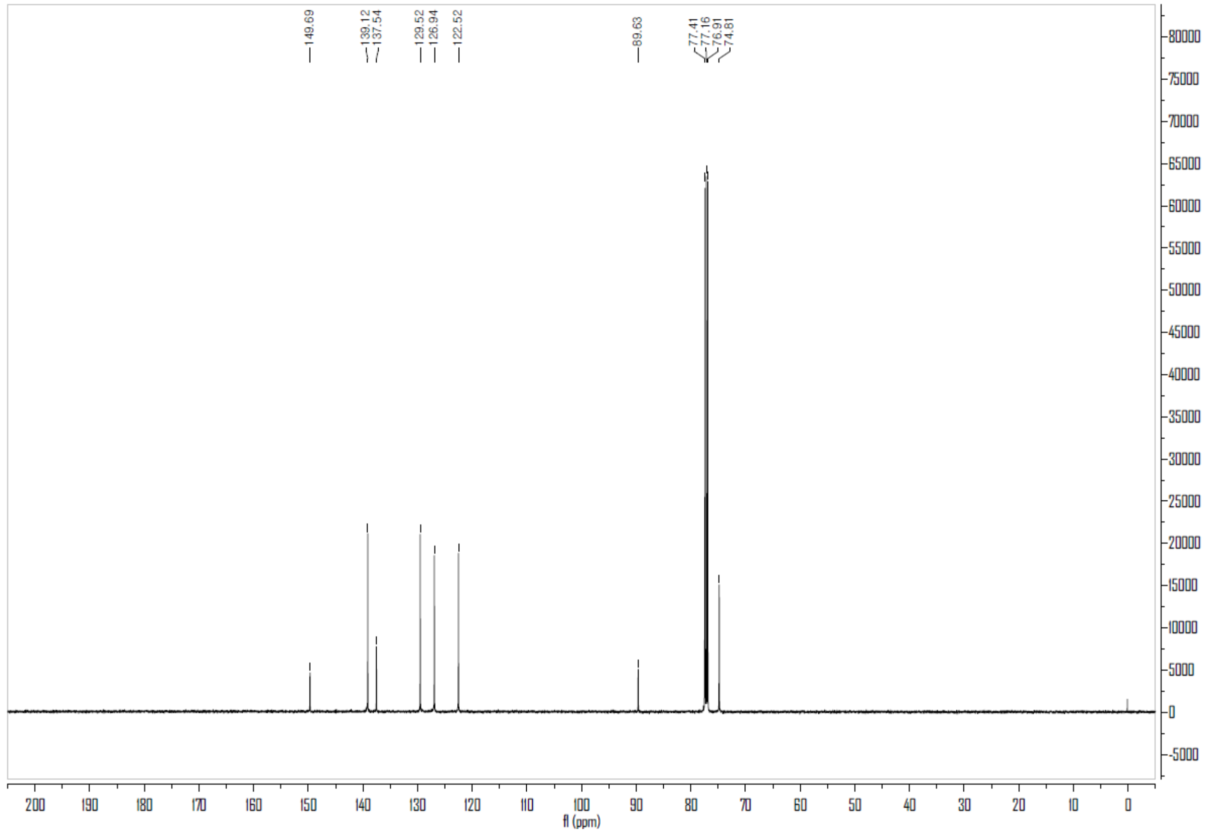

N-(2-Iodophenyl)-N-methylpropiolamide (8)

^{1}H NMR spectrum of 8

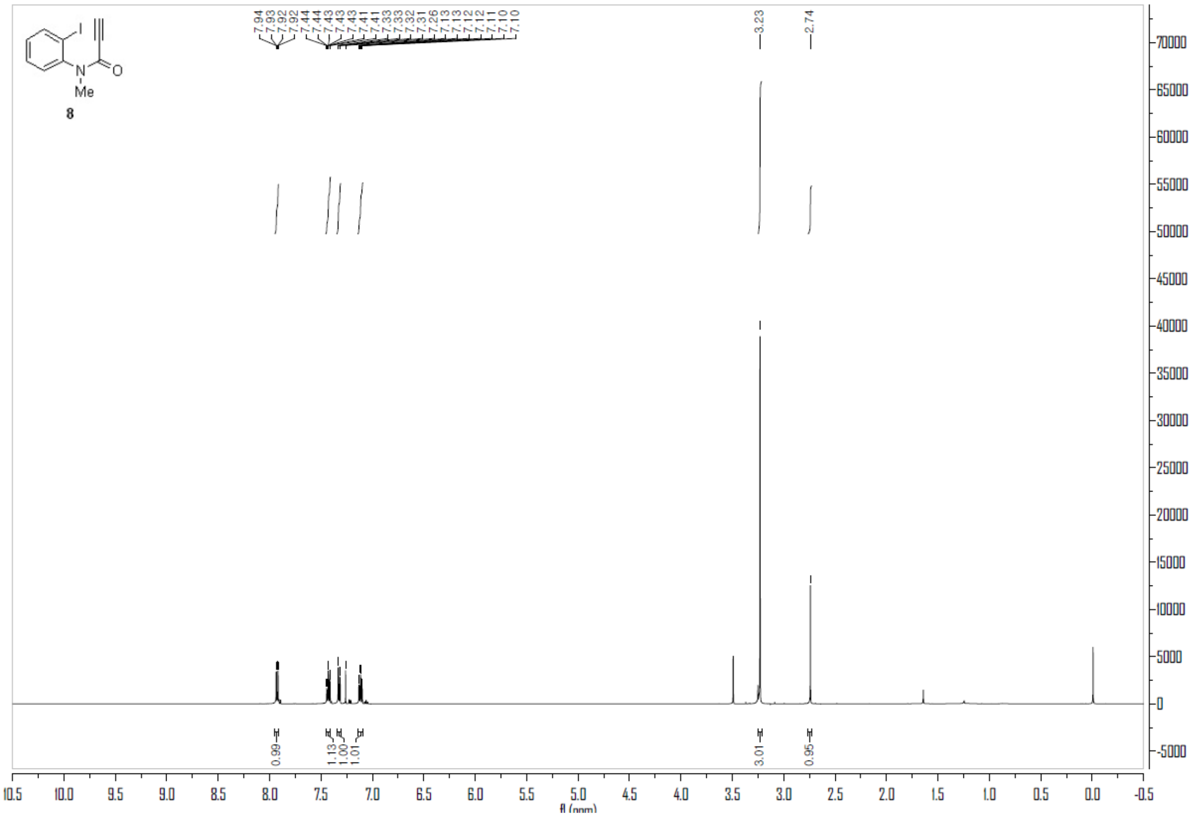

^{13}C NMR spectrum of 8

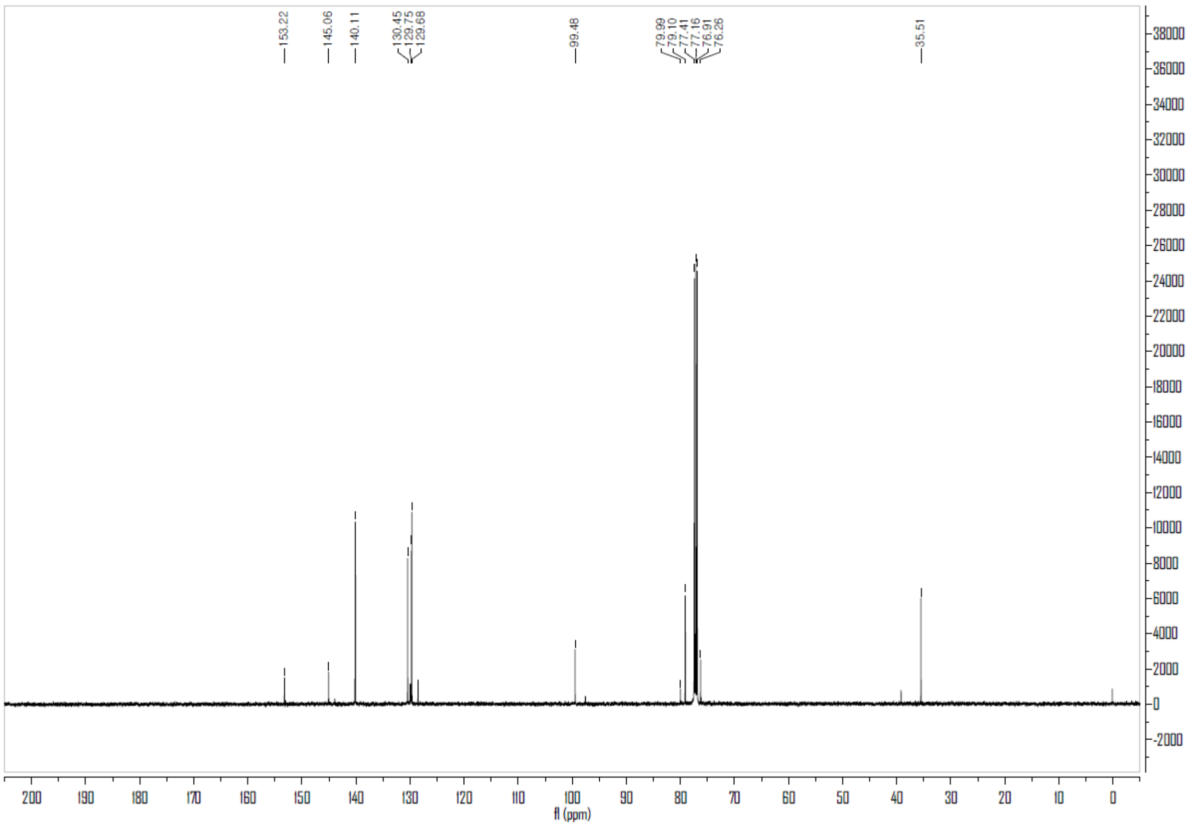

(4-((2-Iodophenyl)(methyl)amino)-4-oxobut-2-ynoic acid (9)

^{1}H NMR spectrum of 9

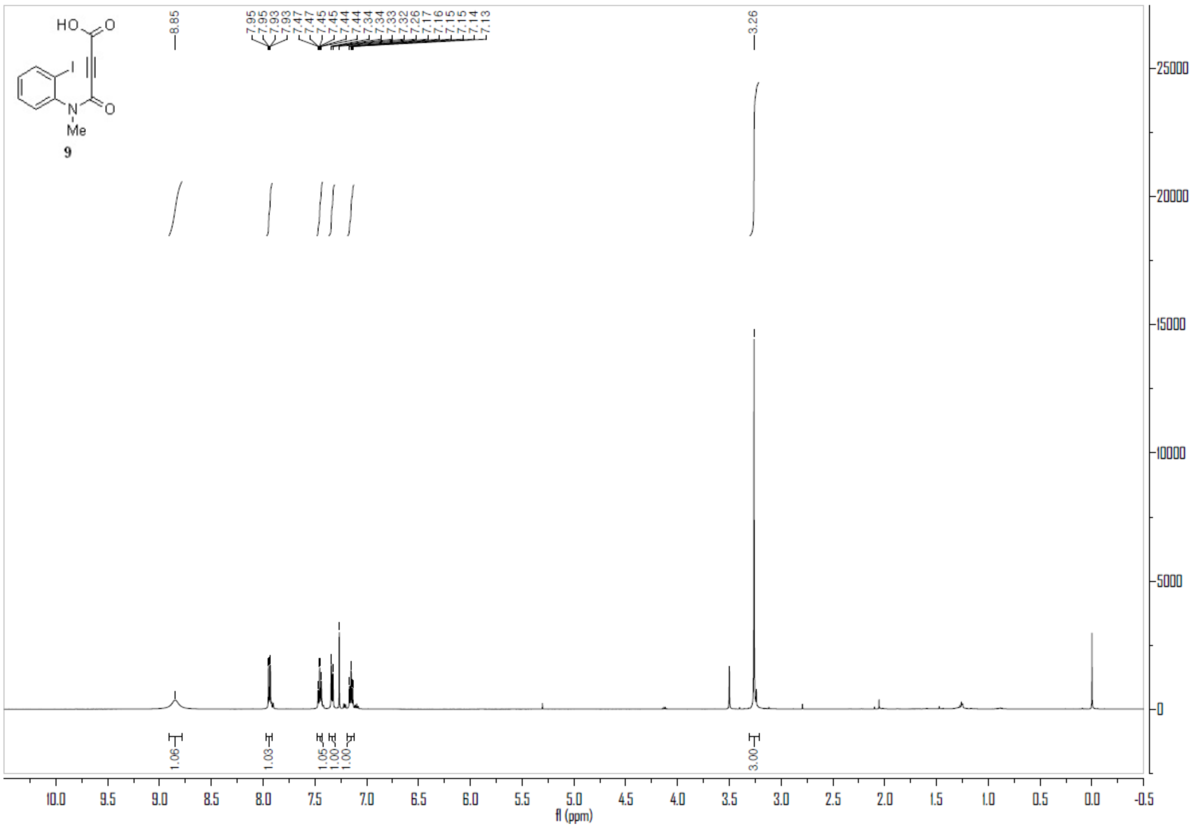

^{13}C NMR spectrum of 9

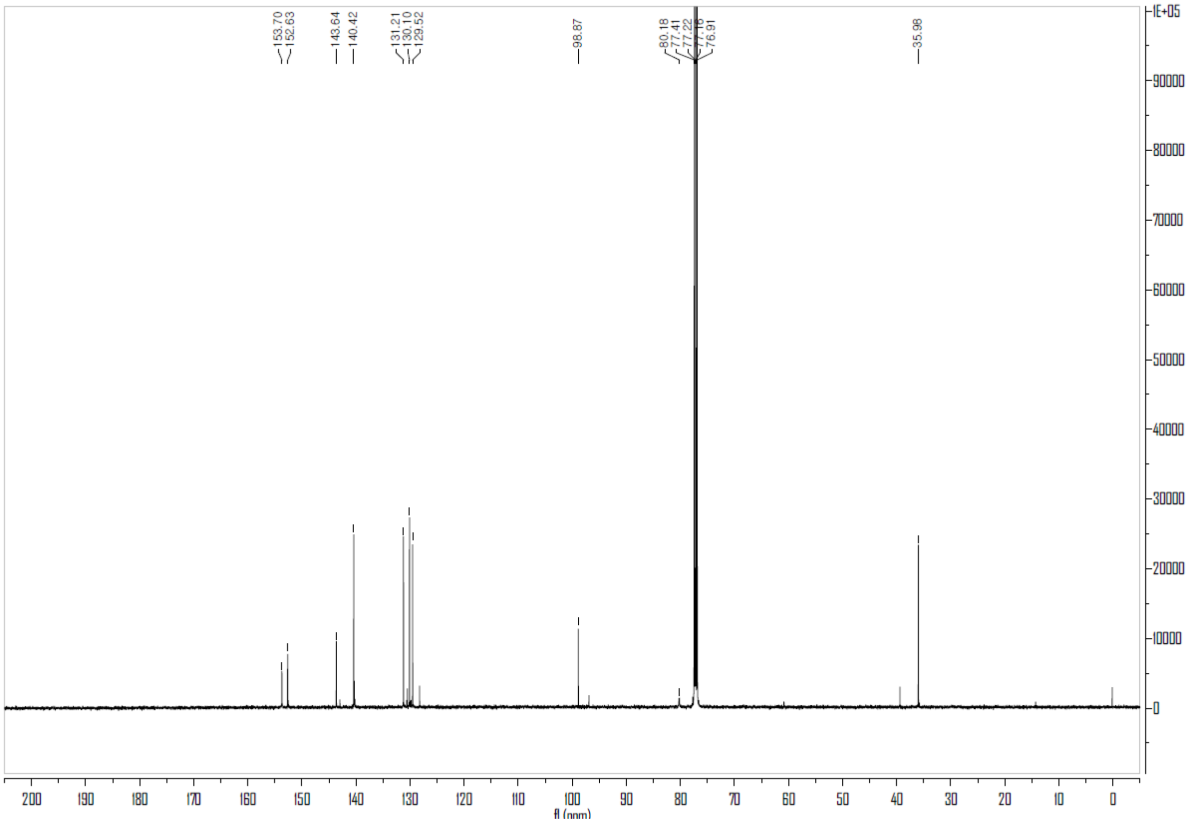

N^1-(2-Iodophenyl)-N^1-methyl-N^4-(2-methylbut-3-yn-2-yl)but-2-ynediamide (2)

^{1}H NMR spectrum of 2

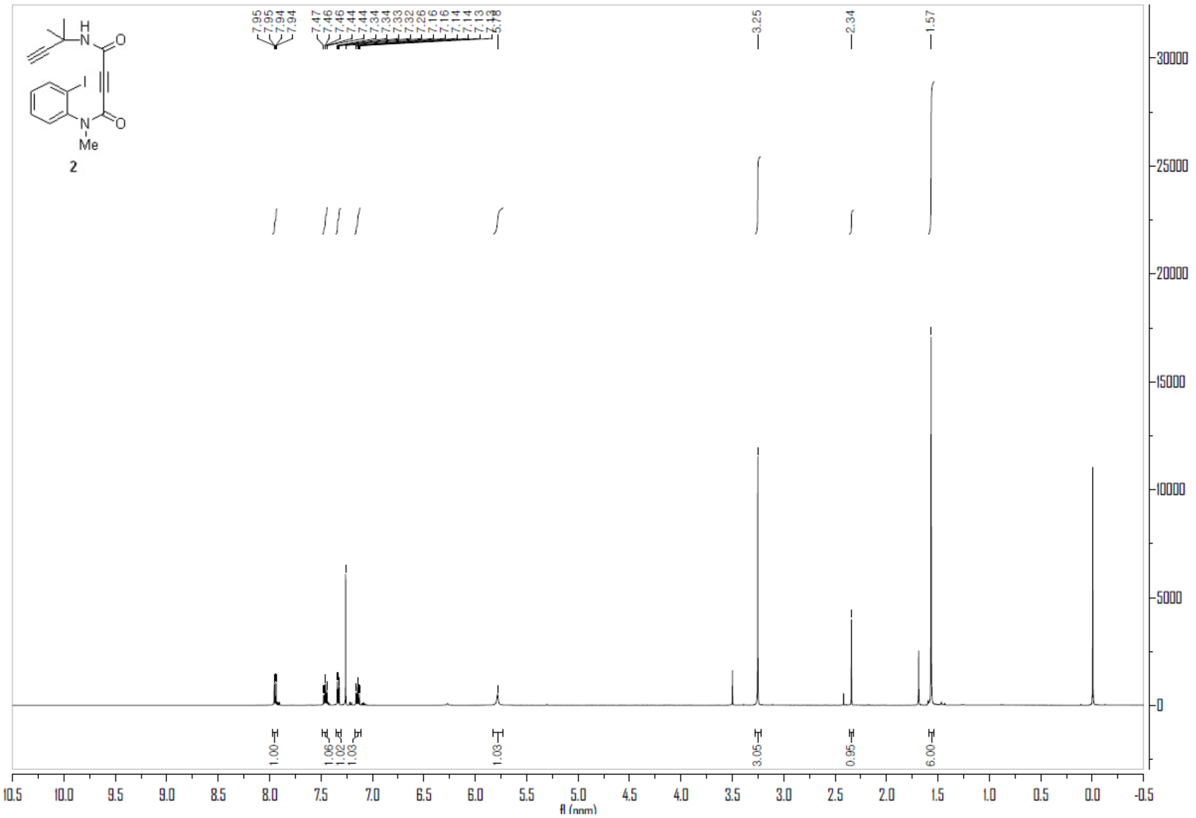

^{13}C NMR spectrum of 3c

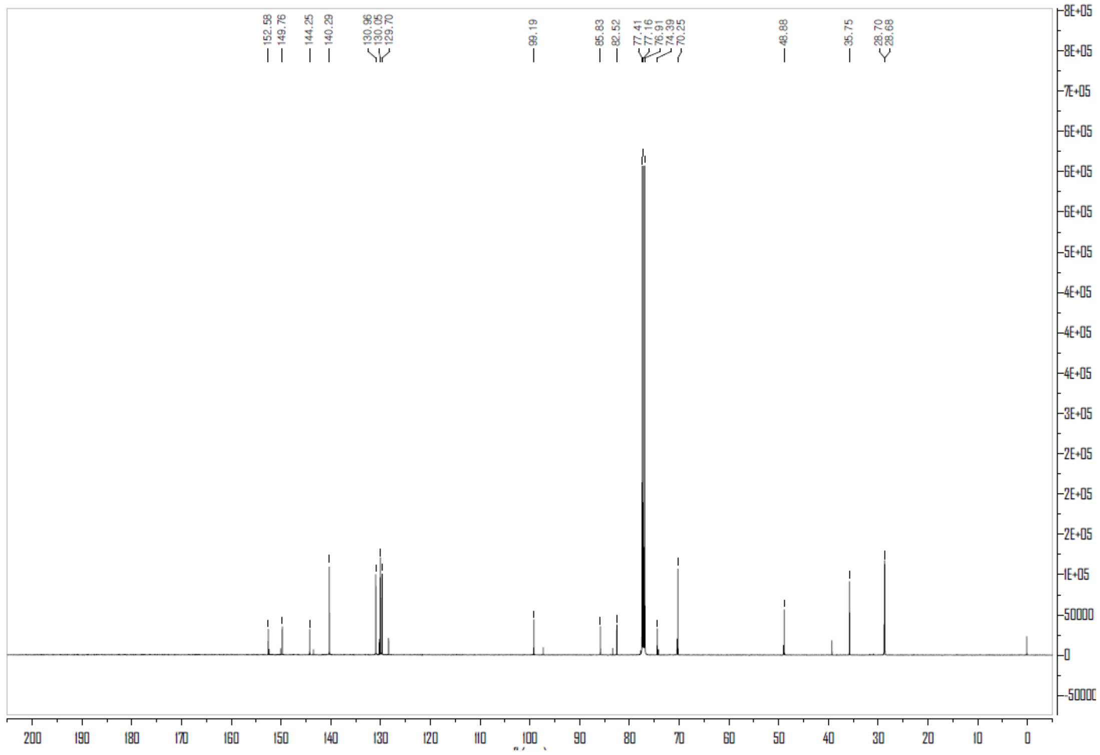

N^1-(4-(tert-Butyldimethylsilyl)-2-methylbut-3-yn-2-yl)-N^4-(2-iodophenyl)-N^4-methylbut-2-ynediamide (10)

^{1}H NMR spectrum of 10

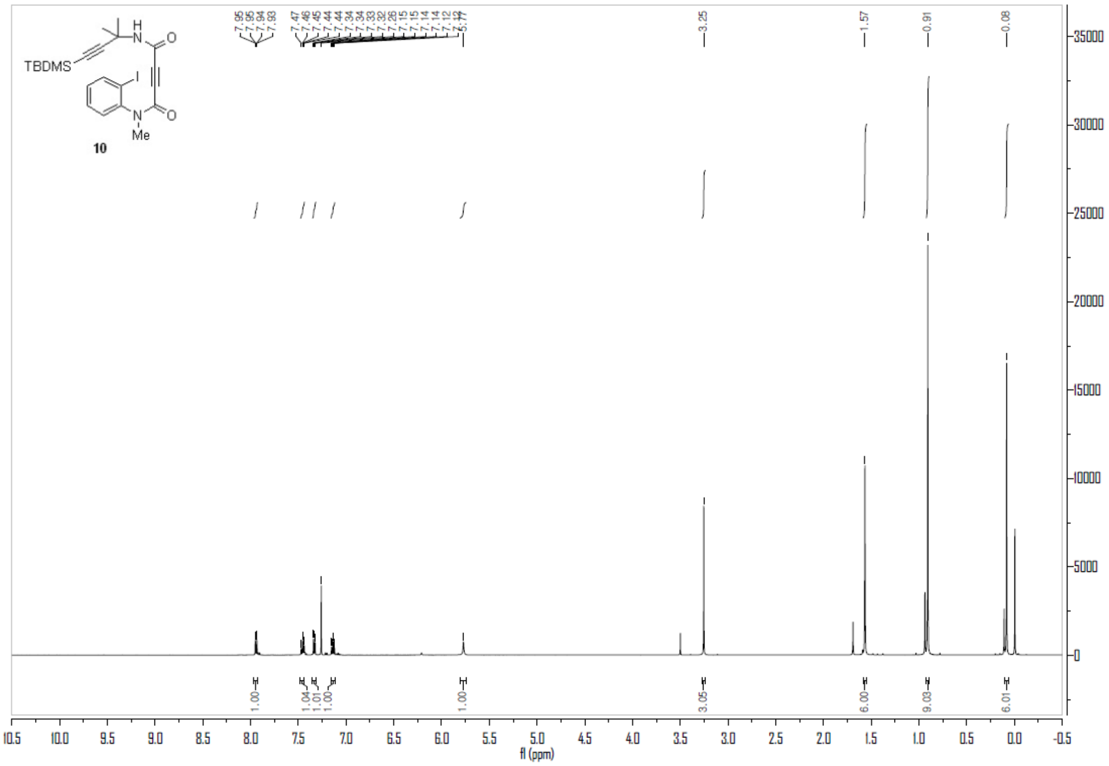

^{13}C NMR spectrum of 10

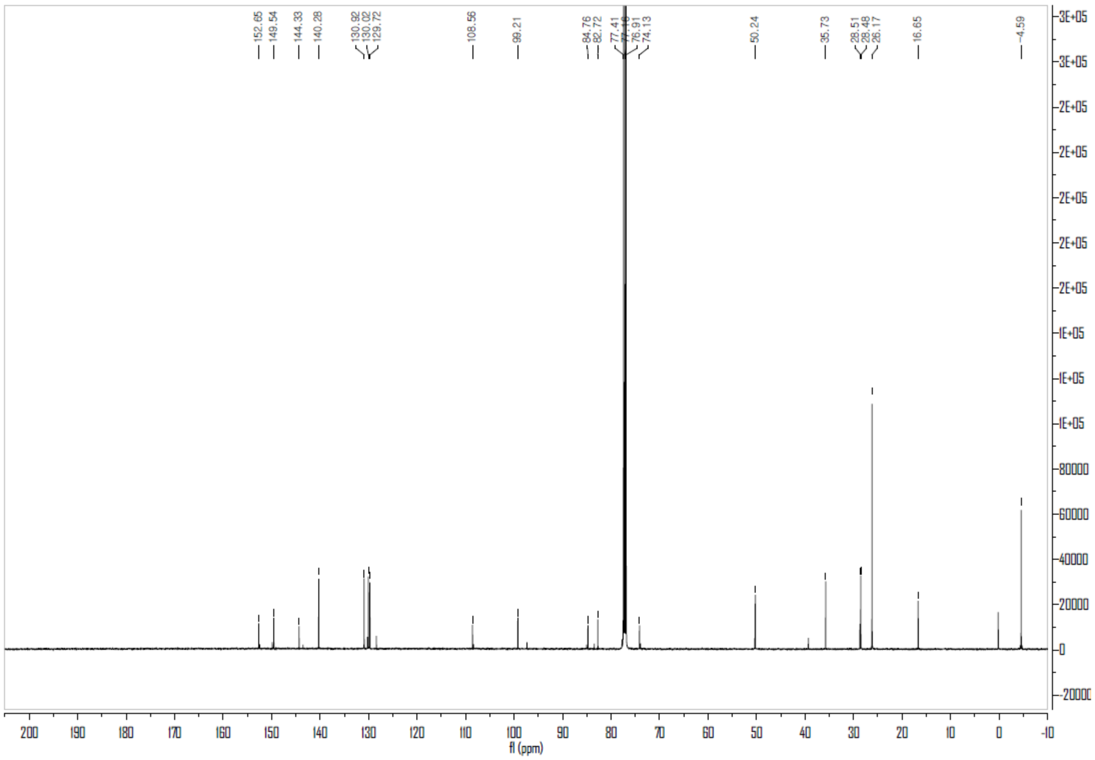

6-(tert-Butyldimethylsilyl)-2,7,7-trimethyl-7,8-dihydro-1H-isoindolo[4,5,6-cd]indole-1,9(2H)-dione (11)

^{1}H NMR spectrum of 11

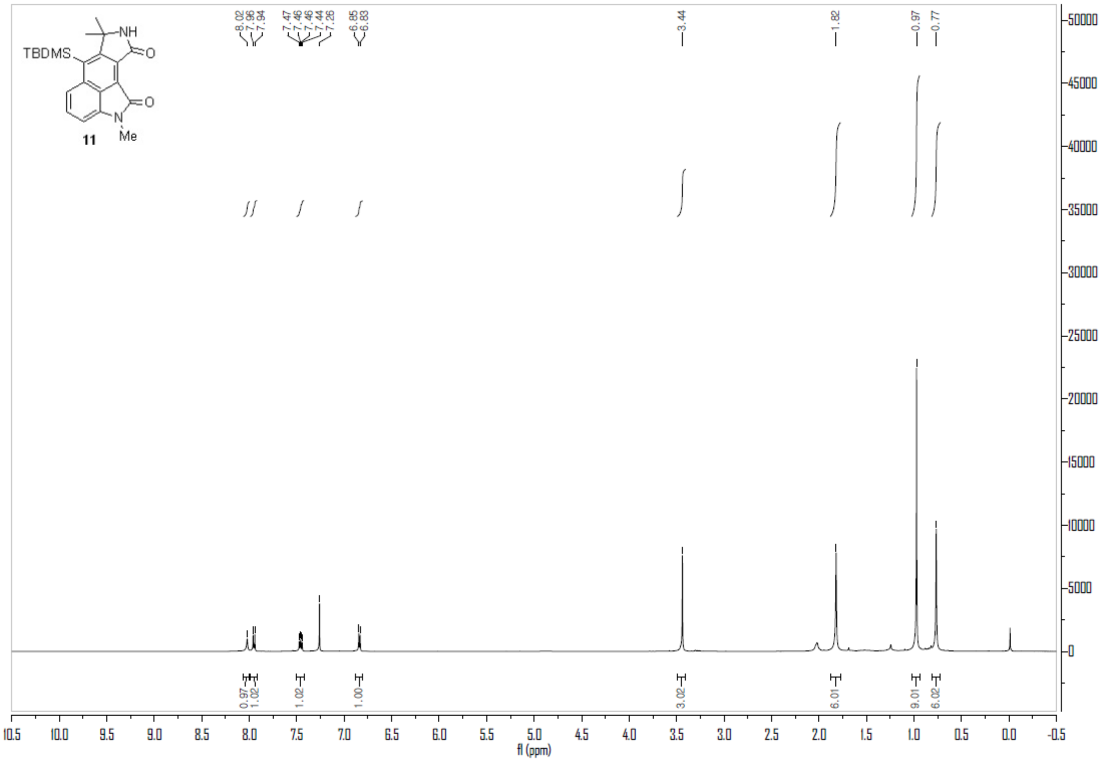

^{13}C NMR spectrum of 11

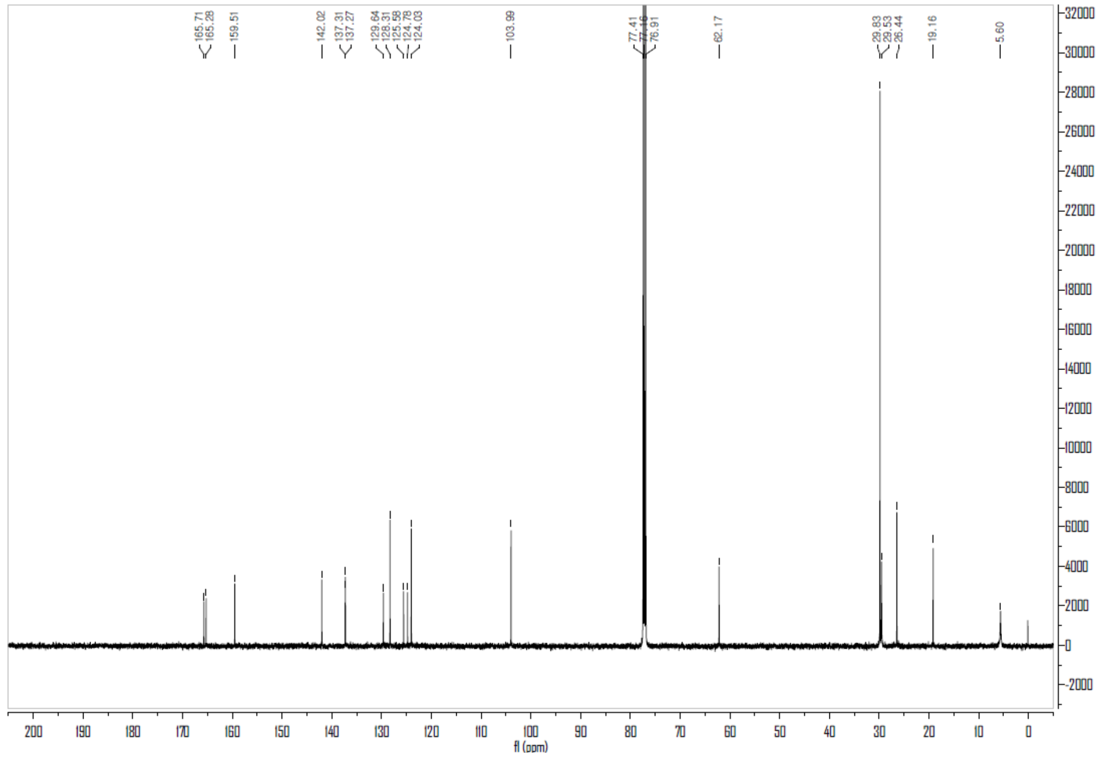

HSQC spectrum of 11

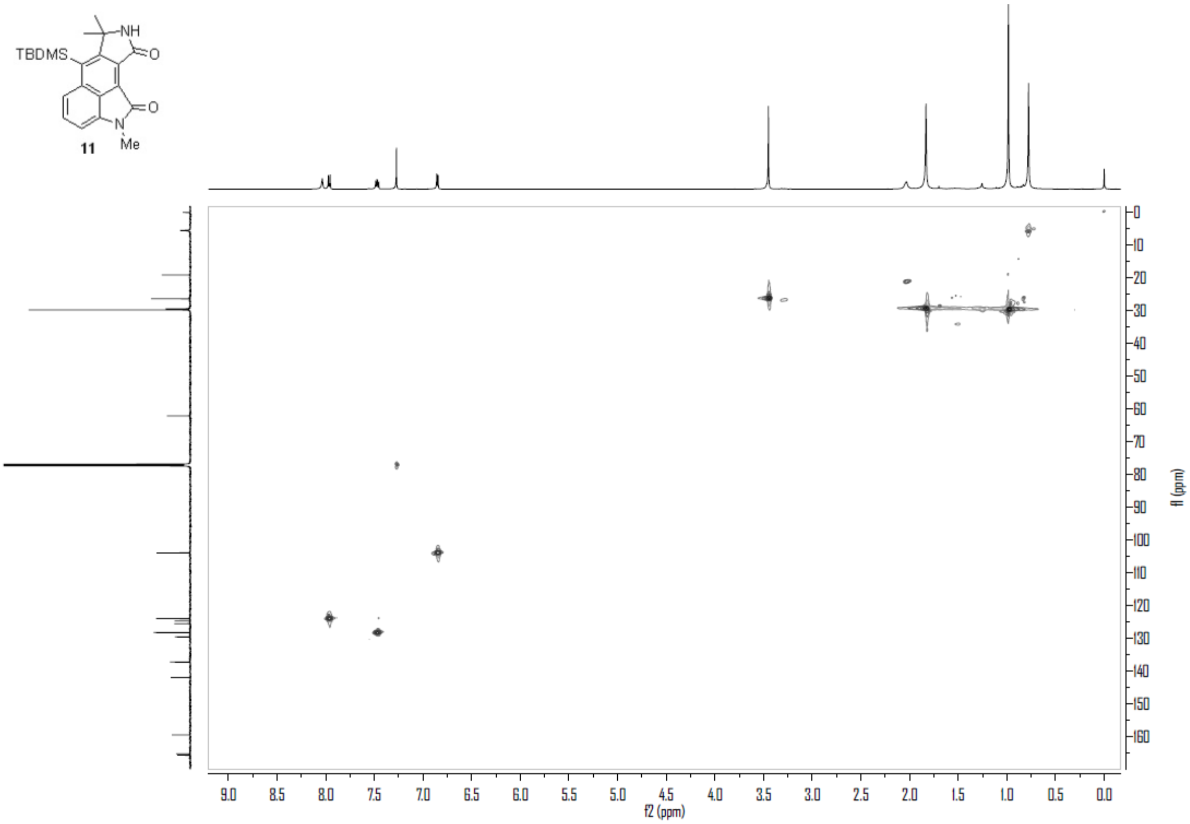

HMBC spectrum of 11

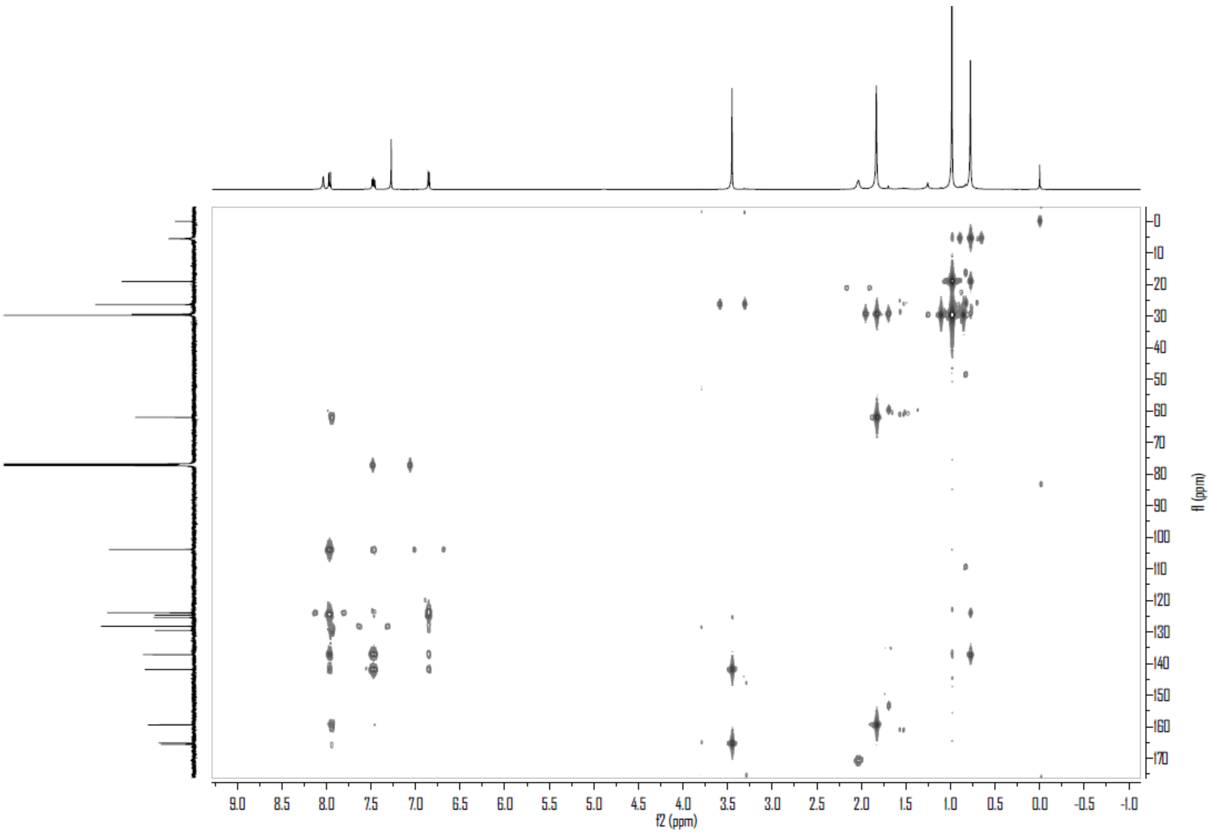

Cyclopiamide A (1)

^{1}H NMR spectrum of 1

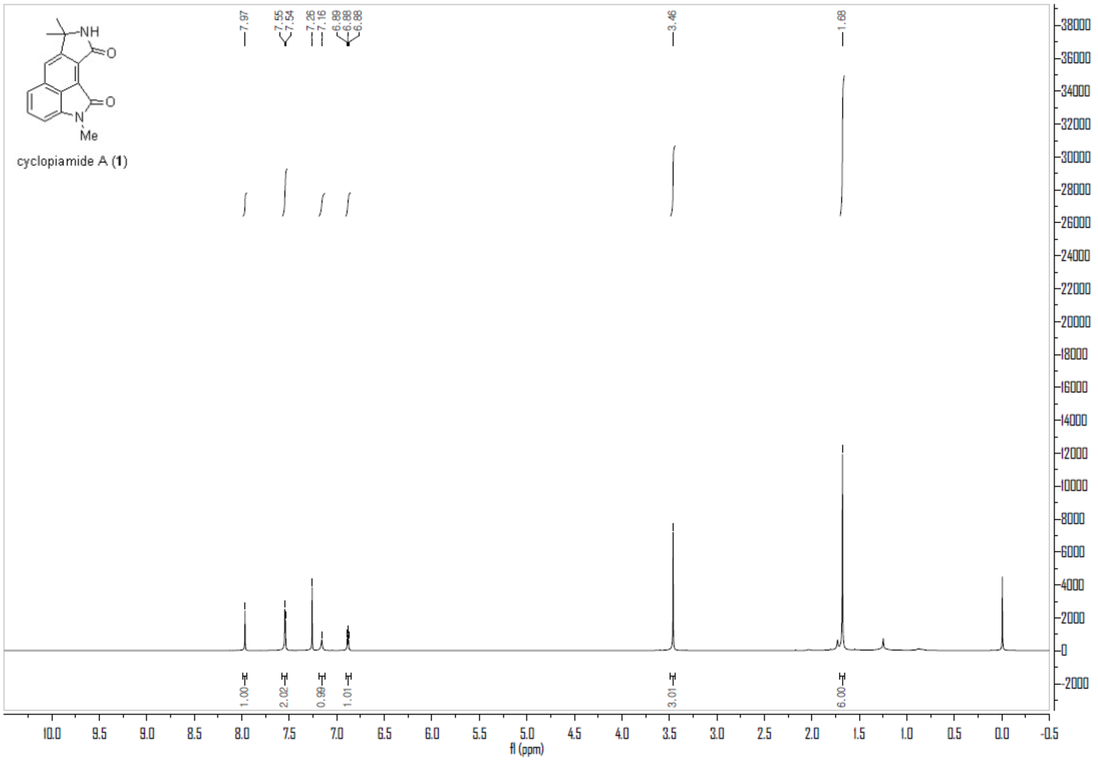

^{13}C NMR spectrum of 1

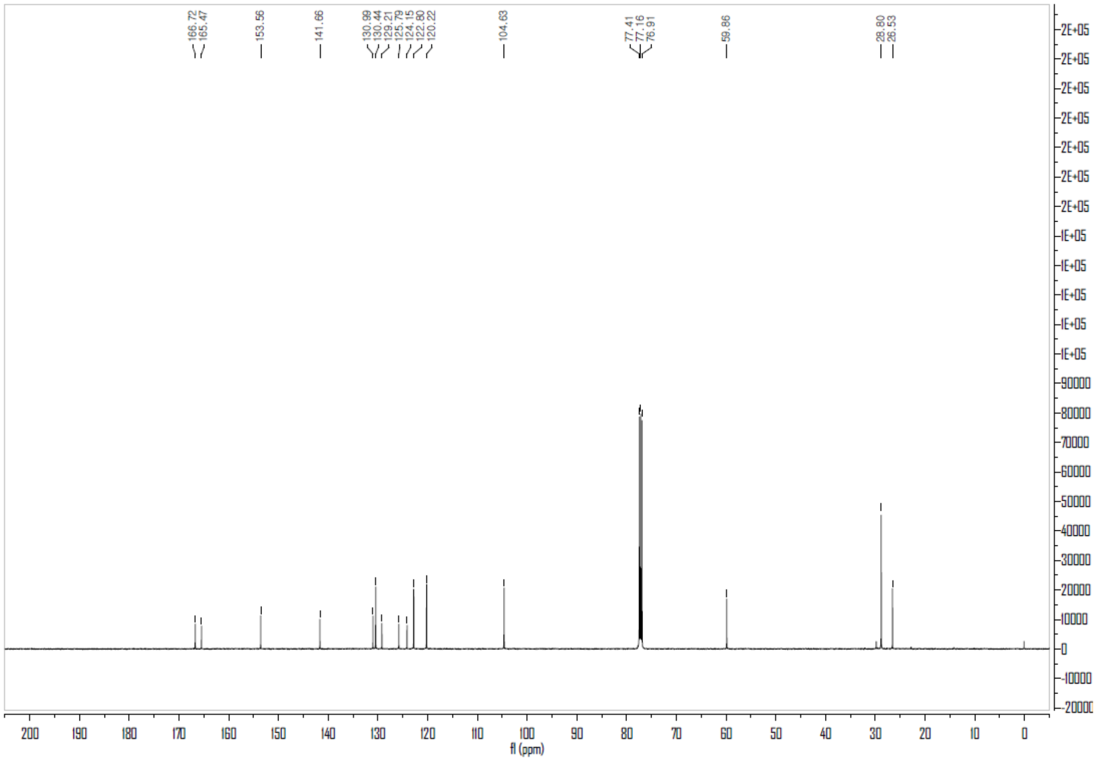

HSQC spectrum of 1

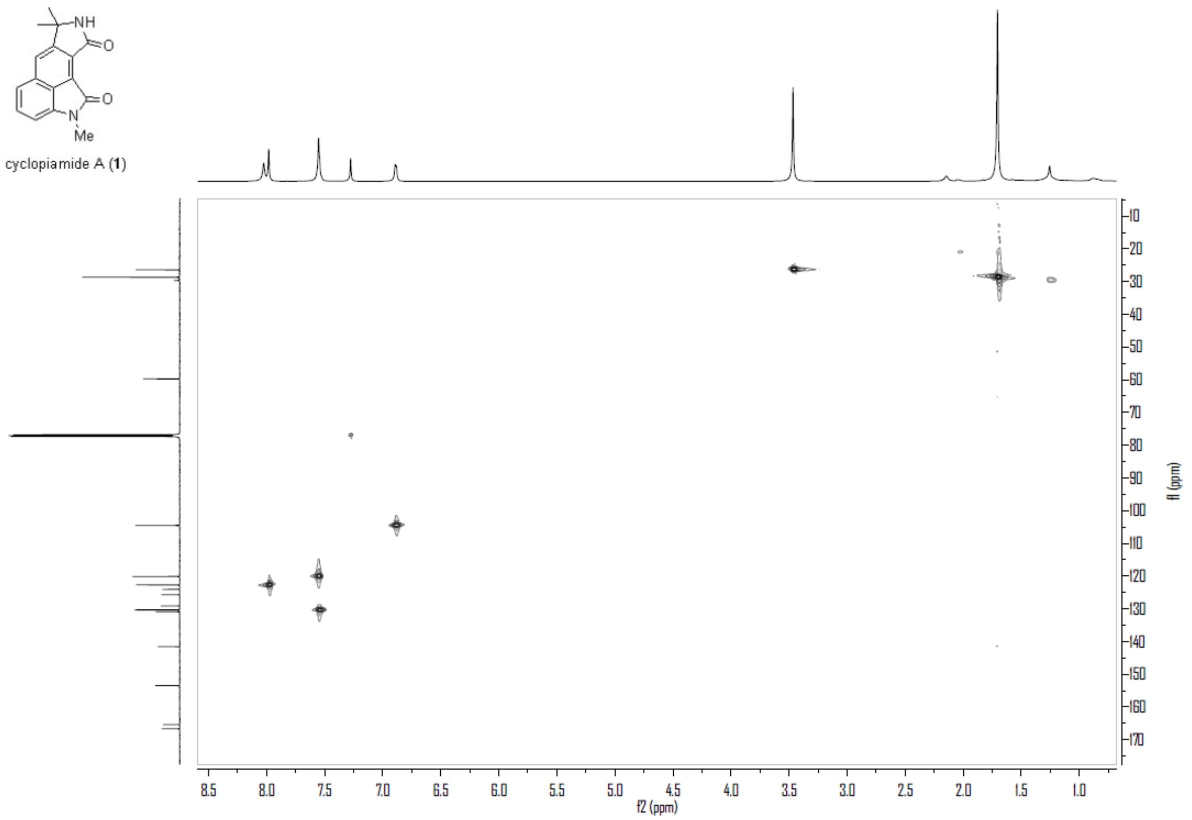

HMBC spectrum of 1

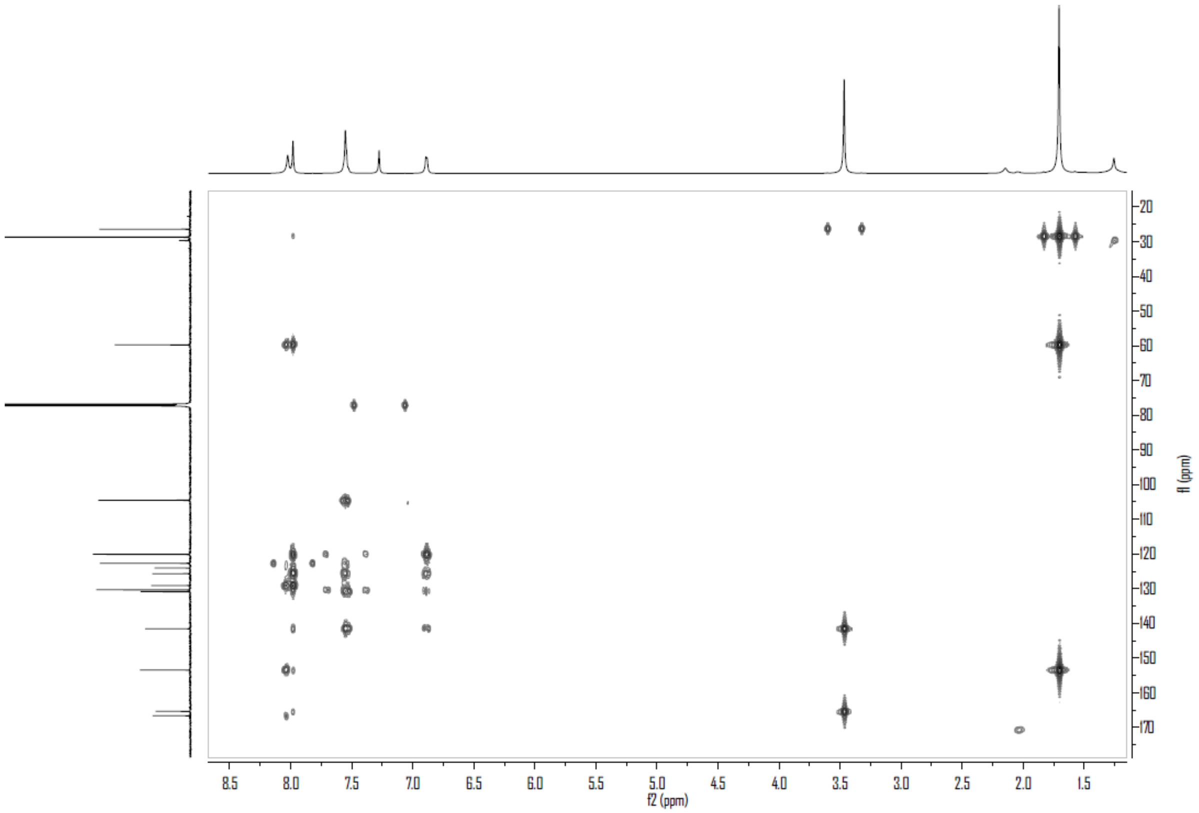

Tabulated spectral comparison of ^{1}H and ^{13}C NMR spectra between synthetic and natural cyclopiamide A

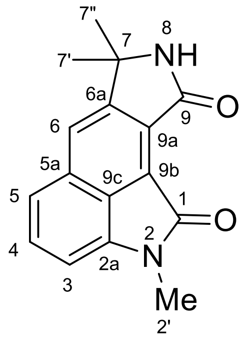

cyclopiamide A (**1**)

^{1}H NMR

H	Isolation data (A)[1]	Our data (B)	Wood group's data[2]	Δ (A-B)
H6	7.94 (s)	7.97 (s)	7.96 (s)	- 0.03
H8	7.82 (brs)	7.16 (brs)	6.32 (brs)	- 0.66
H4 &5	7.51 (m)	7.54 (m)	7.55 (m)	- 0.03
H3	6.85 (dd)	6.88 (t)	6.89 (dd)	+ 0.03
H2'	3.41 (s)	3.46 (s)	3.46 (s)	+ 0.05
H7' & 7"	1.66 (s)	1.68 (s)	1.66 (s)	- 0.02

Deviation of H8 data might be due to amide N-H character of H8

^{13}C NMR

C	Isolation data (A)[1]	Our data (B)	Wood group's data[2]	Δ (A-B)
C9	166.5	166.7	166.3	- 0.2
C1	165.4	165.5	165.4	- 0.1
C6a	153.4	153.6	153.3	- 0.2
C2a	141.4	141.7	141.7	- 0.3
C9b	130.8	131.0	131.0	- 0.2
C4	130.3	130.4	130.6	- 0.1
C9a	129.0	129.2	128.9	- 0.2
C9c	125.6	125.8	125.8	- 0.2
C5a	124.0	124.2	124.3	- 0.2
C6	122.7	122.8	122.8	- 0.1
C5	120.1	120.2	120.2	- 0.1
C3	104.6	104.6	104.7	0
C7	59.8	59.9	59.7	- 0.1
C7' & 7"	28.6	28.8	28.9	- 0.2
C2'	26.4	26.5	26.6	- 0.1

[1]Holzapel, C.W.; Bredenkamp, M.W.; Snyman, R.M.; Boeyenes, J.C.A.; Callen, C. Cyclopyamide, an Isoindolo[4,6-cd]indole from *Penicillium Cyclopium*. *Phytochemistry* **1990**, *29*, 639–642.

[2]Nakhla, M.C.; Weeks, K.N.; Villalobos, M.N.; Wood, J.L. Total Synthesis of Cyclopiamide A and Speradine E. *Tetrahedron* **2018**, *74*, 5085–5088.
